# Evaluating ginkgetin from *Ginkgo biloba* as a novel agent for sleep promotion through molecular docking and *in vivo* studies

**DOI:** 10.22038/ijbms.2025.82718.17878

**Published:** 2025

**Authors:** Mir Behrad Aghazadeh Ghadim, Ebrahim Salimi-Sabour, Alireza Shahriari, Mahdi Niazi, Farideh Bahrami

**Affiliations:** 1 Neuroscience Research Center, Baqiyatallah University of Medical Sciences, Tehran, Iran; 2 Department of Physiology and Medical Physics, School of Medicine, Baqiyatallah University of Medical Sciences, Tehran, Iran; 3 Department of Pharmacognosy and Traditional Pharmacy, Faculty of Pharmacy, Baqiyatallah University of Medical Sciences, Tehran, Iran; 4 Chemical Injuries Research Center, Systems Biology and Poisonings Institute, Baqiyatallah University of Medical Sciences, Tehran, Iran

**Keywords:** GABAA receptor, Ginkgo biloba, Ginkgetin, Molecular docking, Sleep

## Abstract

**Objective(s):**

Sleep impacts the well-being and quality of life of millions. Given conventional pharmacotherapy’s limitations and side effects, the quest for adequate and proper sleep promotion is imperative. This study aims to identify a suitable and effective compound for sleep by examining qualified herbal compounds in the PubChem database using *in silico* methods. Ultimately, the extracted compound (ginkgetin, a bioactive flavonoid from *Ginkgo biloba*) through molecular docking by considering the GABAA receptors will be evaluated through the *in vivo *method in an animal model to serve as proof for the findings from the molecular docking process.

**Materials and Methods:**

Utilizing a comprehensive approach, this research employed molecular docking to screen 2299 phytochemicals for their affinity towards the GABAA receptor, focusing on the GABA, benzodiazepine, and steroid-binding sites. Ginkgetin emerged as a top candidate due to its high binding affinity. Subsequent* in vivo *electrophysiological assessments in rats treated with *G. biloba *extract containing ginkgetin evaluated alterations in sleep architecture, REM, and NREM sleep phases.

**Results:**

Molecular docking identified ginkgetin as possessing the highest binding affinity among the screened phytochemicals.* In vivo* studies corroborated these findings, demonstrating that rats treated with *Ginkgo biloba *extract significantly enhanced REM and NREM sleep compared to controls.

**Conclusion:**

Ginkgetin, derived from *G. biloba*, shows promising potential as a novel therapeutic agent for sleep disorders, supported by its strong affinity to key receptor sites and its efficacy in modulating sleep architecture* in vivo*. These findings contribute to the expanding evidence base for the therapeutic use of *G. biloba* in sleep promotion and underscore the need for further research to elucidate the mechanisms and clinical applicability of ginkgetin in sleep disorder treatment.

## Introduction

The γ-aminobutyric acid type A receptor (GABAA receptor) is a cornerstone of inhibitory neurotransmission in the central nervous system, playing a pivotal role in maintaining the delicate balance between neuronal excitation and inhibition (1). This balance is crucial for normal brain function, and disruptions can lead to a plethora of neurological and psychiatric disorders. The GABAA receptor, a ligand-gated ion channel, is instrumental in mediating the inhibitory effects of GABA, the primary inhibitory neurotransmitter in the brain. Dysfunctions in GABAA receptor signaling have been implicated in the pathophysiology of various conditions, including anxiety disorders, major depressive disorder, and epilepsy, highlighting its significance as a therapeutic target (2-4). The quest for novel therapeutic agents has increasingly turned towards the natural world, with phytochemicals emerging as a rich source of bioactive compounds. These naturally occurring substances, which have evolved over millennia, offer a diverse chemical space for identifying novel modulators of biological targets. The therapeutic potential of phytochemicals is underpinned by their historical use in traditional medicine and their demonstrated efficacy in modulating key molecular pathways involved in disease processes (5). Among these, the modulation of GABAA receptor function by specific phytochemicals has garnered significant attention, offering a promising avenue for developing new anxiolytic and anticonvulsant therapies (6-9). Molecular docking, a computational technique at the forefront of drug discovery, enables the prediction of the interaction between a small molecule (ligand) and a protein (receptor) at the atomic level (10). By simulating the possible orientations of the ligand within the receptor’s binding site, molecular docking provides insights into the binding affinity and the most energetically favorable conformation of the ligand-receptor complex. This technique is particularly valuable for screening large libraries of compounds, such as phytochemicals, to identify those with the potential to modulate target proteins effectively. The accuracy and efficiency of molecular docking have made it an indispensable tool in the early stages of drug development, guiding the selection of candidates for further experimental validation (11). In light of the critical role of the GABAA receptor in neurological function and the potential of phytochemicals as sources of neurophysiological agents, our study aims to comprehensively evaluate the modulatory effects of over two thousand phytochemicals on the GABAA receptor. We utilize advanced molecular docking techniques to identify promising phytochemicals that exhibit strong binding affinity and favorable interaction profiles with the GABAA receptor. Additionally, the effects of the selected component, as indicated by the result of the in-silico study, are evaluated through an *in vivo *study in rats, where the animals’ sleep levels are measured.

## Materials and Methods

### 3D structures preparation

*Phytochemical library compilation*

Our investigation leveraged an extensive phytochemical library sourced from the Kyoto Encyclopedia of Genes and Genomes (KEGG) database (12). This curated collection encompassed various chemical scaffolds, totaling 2845 distinct compounds. The library’s composition was stratified into several categories based on chemical nature and biological origin: Alkaloids (714 compounds), Amino Acid-Derived Compounds (104), Fatty Acid-Derived Compounds (37), Flavonoids (478), Phenylpropanoids (189), Polyketides (136), Compounds Derived from the Shikimate/Acetate-Malonate Pathway (45), Terpenoids (101), and an additional category encompassing various other compounds (41).

*3D Structure acquisition of phytochemicals*

The phytochemicals’ three-dimensional (3D) structures were meticulously obtained from the PubChem database, a reputable repository for chemical information (https://pubchem.ncbi.nlm.nih.gov). Each compound’s 3D conformation was carefully selected to ensure the highest level of structural fidelity and relevance for subsequent computational analyses.

*GABAA receptor structure retrieval*

The structural basis for our molecular docking studies was the 3D crystal structures of the GABAA receptor, which were retrieved from the Protein Data Bank (PDB) (https://www.rcsb.org/). The selection of receptor structures was comprehensive, including multiple conformations to adequately represent the receptor’s structural diversity and potential conformational states relevant to ligand binding. The specific PDB entries utilized in our study were 6CDU, 6D1S, 6D6T, 6D6U, 6HUG, 6HUJ, 6HUK, 6HUO, 6HUP, 6I53, 6X3S, 6X3T, 6X3U, 6X3V, 6X3W, 6X3X, 6X3Z, and 6X40. These structures were selected based on their resolution, completeness, and relevance to the GABAA receptor’s pharmacologically active sites.

### SwissADME analysis

*Evaluation of drug-like properties*

To ascertain the drug-likeness of the 2845 phytochemicals within our library, we employed the SwissADME web tool (http://www.swissadme.ch), a comprehensive resource for assessing molecular compounds’ pharmacokinetic and drug-like characteristics. This analysis is pivotal in early-stage drug discovery for filtering compounds with favorable oral bioavailability and permeation characteristics.

*Application of Lipinski’s rule of five*

Lipinski’s “Rule of Five” was the cornerstone of our drug-likeness assessment. This rule provides a heuristic to evaluate the drug-likeness of compounds based on their physicochemical properties, serving as an initial filter to identify molecules with suitable attributes for oral bioavailability. According to Lipinski’s criteria, a compound is considered to possess drug-like properties if it meets the following conditions: a molecular weight (MW) under 500 Dalton, a Consensus logP (CLogP) not exceeding 5, no more than five hydrogen bond donors (HBD), and ten or fewer hydrogen bond acceptors (HBA). These parameters indicate a compound’s permeation and absorption potential, crucial factors for oral administration efficacy (13, 14).

### Molecular docking

*Preparation of GABAA receptor structures*

The initial step in our molecular docking protocol involved meticulously preparing the GABAA receptor structures for docking simulations. This preparation included the removal of water molecules and co-crystallized ligands from the receptor structures to ensure a clean binding site for docking. The receptor structures were then processed using MGLTools 1.5.6 (15), which facilitated the conversion of the structures into the PDBQT format, essential for docking simulations. This format includes the atomic coordinates, partial charges, and atom types, with Gasteiger partial charges added to the receptor atoms to model electrostatic interactions accurately.

*Docking protocol validation*

We employed a self-docking strategy to ensure the reliability and accuracy of our docking simulations. This involved re-docking the co-crystallized ligands back into their respective binding sites on the receptor, aiming to reproduce the known ligand-receptor interactions as closely as possible. The validation criterion for the docking protocol was the achievement of root-mean-square deviation (RMSD) values of less than 2 Å, indicating a high degree of congruence between the docked conformation and the original ligand orientation within the crystal structure.

*Docking simulations*

For each receptor structure, the docking grid was centered on the coordinates of the central atom of the co-crystallized ligand, ensuring the focus was on the biologically relevant binding site. The dimensions of the docking grid were set to 30x30x30 Å to adequately encompass the entire binding site. The docking simulations were executed with an exhaustiveness parameter of 8 to balance computational efficiency with the thoroughness of the search, using AutoDock Vina (16) on an eight-core computational system for enhanced performance.

*Verification and further analysis with autoDock4*

Following the initial docking simulations conducted with AutoDock Vina, further validation and in-depth analysis of the docking results were performed using AutoDock4 (AD4), version 4.2.6 (17). This step was crucial for corroborating AutoDock Vina’s findings and leveraging AD4’s distinct scoring functions and search algorithms to gain additional insights into the ligand-receptor interactions.

*Grid preparation and docking parameters*

The preparation of grid maps, a critical component of the docking process in AD4, was executed using the AutoGrid program (17), an integral part of the AD4 suite. For the receptor with PDB code 6X3S, the grid box dimensions were uniformly set to 30 Å in the x, y, and z axes to encompass the entire binding site. The spacing between grid points was maintained at 1.0 Å, ensuring a fine resolution for accurately capturing the potential interactions between the ligands and the receptor. The grid center was meticulously positioned at coordinates 115.387 Å, 109.954 Å, and 154.697 Å for the x, y, and z axes, respectively, aligning with the geometric center of the binding site to ensure the focal area of the docking simulations was the biologically relevant interaction zone.

*Lamarckian genetic algorithm and docking execution*

The docking simulations within AD4 were driven by the lamarckian genetic algorithm (LGA), renowned for its efficacy in identifying optimal conformations of ligand-receptor complexes. This algorithm combines the principles of Darwinian evolution with the concept of phenotype-to-genotype learning, allowing for an efficient exploration of the conformational space. A maximum of 10 conformers were evaluated for each ligand to identify the most energetically favorable binding poses. The docking runs adhered to the default parameters set by AD4, which included a population size of 150, a maximum of 2,500,000 energy evaluations, and 27,000 generations, ensuring a comprehensive search. The settings also specified the automatic survival of the top individual from each generation, a gene mutation rate of 0.02, and a crossover rate of 0.8, optimizing the balance between exploration and exploitation in the search algorithm.

### Preparation of G. biloba plant leaves and extraction

*Source and preparation of plant material*

The leaves of the *G. biloba* plant were sourced as dried specimens from the National Botanical Garden of Iran, along with a high-quality and authenticated sample imported from China. Nagoya protocol and sterility have been considered in the preparation of the extract. The product is manufactured and used at the laboratory level. Before extraction, the leaves were subjected to further drying to ensure optimal moisture content. This was conducted over one week in a controlled environment shielded from direct sunlight and maintained at ambient room temperature to preserve the integrity of the phytochemical constituents.

*Extraction procedure*

The extraction of bioactive compounds from the dried *G. biloba* leaves was performed using the percolation method, a technique renowned for its efficiency in extracting soluble plant constituents (18). Initially, 100 grams of finely powdered *G. biloba* leaves were placed into a percolator. This was followed by adding a solvent mixture of methanol and water in an 8:2 ratio, selected for its efficacy in solubilizing a wide range of phytochemicals. The plant material was allowed to macerate in this solvent for 24 hr, facilitating the thorough leaching of soluble compounds.

*Fractionation and concentration*

After the percolation process, the resulting extract was carefully collected during the gradual extraction. This procedure was repeated four times with fresh solvent. The collected extracts were then subjected to a concentration process using a rotary evaporator. The conditions were meticulously set to a temperature of 40 degrees Celsius and a rotation speed of 100 rpm under reduced pressure to gently remove the solvent without compromising the stability of the sensitive phytochemicals. 

*Solvent extraction method*

This step involved sequentially partitioning the concentrated extract with solvents of varying polarities, allowing for the selective separation of compounds based on their solubility and chemical affinity. This fractionation process was instrumental in obtaining distinct phytochemical profiles for subsequent analysis and testing (19).

*High-performance thin layer chromatography*

**Figure 1:**
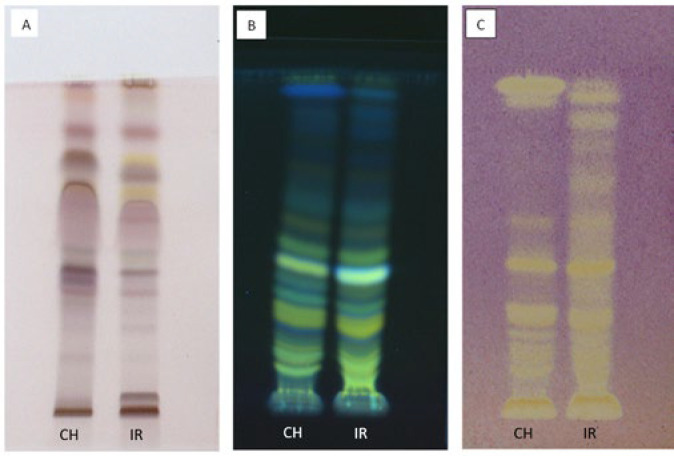
Analysis method: High-performance thin layer chromatography

Two Iranian and Chinese extract samples were subjected to high-performance thin-layer chromatography analysis to display terpenes, flavonoids, phenolic acid, and anti-oxidant compounds. The results are shown in Figure 1.

### Treatment of animals with G. biloba alcohol extract

*Animal selection and grouping*

In this *in vivo* study, twenty-one Wistar rats were selected. The animals were acclimatized to laboratory conditions for one week prior to the commencement of the treatments, with free access to food and water, and maintained on a 12-hr light/dark cycle. The rats were then randomly divided into three groups to assess the effects of the Chinese type of *G. biloba* alcohol extract (via oral gavage) at a dose of 200 mg/kg body weight (n=7) and compare them with a control group received solvent (n=7) and a group treated with Valerian extract known anxiolytic and sedative properties, providing a basis for evaluating the effects of the *G. biloba* extract, at a dose of 400 mg/kg body weight (n=7).

*Treatment duration and post-surgical care*

The treatment regimen for all groups lasted 21 days, a duration selected to allow for the accumulation of effects from the extracts. Following this pre-treatment period, the rats underwent a surgical procedure to implant electrodes. After surgery, the animals were allowed a recovery period of one week, during which the administration of the extracts and solvent continued to maintain consistent exposure levels. This post-surgical care was meticulously monitored to ensure the well-being of the animals and the reliability of the study’s findings.

### Electrophysiological recording of sleep

The animals were sedated with a precise combination of Ketamine (65 mg/kg) and Xylazine (15 mg/kg) in compliance with ethical guidelines. The stereotaxic apparatus was used, and EEG recordings were taken from three stainless steel electrodes implanted into the skull; EMG activity was simultaneously recorded from the neck muscles using two Teflon-coated electrodes to monitor muscle tone. All electrodes were securely anchored with dental acrylic to ensure stable signal transmission, and the data was collected via a connector placed on the skull. Electrophysiological recordings were conducted over three consecutive days, with each session lasting three hours for both the treatment and control groups.

The electrophysiological signals were captured using the Ruby Mind electrophysiology recording system, a state-of-the-art setup renowned for its precision and reliability. Operating from its base in New York, US, this system provided high-fidelity recordings at a sampling rate of 1000 Hz, ensuring the capture of nuanced electrophysiological phenomena. The EEG signals underwent filtering between 0.5-40 Hz to isolate brain wave frequencies pertinent to sleep stages, while EMG signals were filtered within the 15-500 Hz range to reflect muscle activity accurately. 

### Semi-automatic sleep scoring

*Initial data segmentation and sample selection*

The intricate process of sleep stage classification commenced with segmenting the EEG and EMG recordings into discrete epochs. This initial step involved carefully selecting three distinct and representative samples for each primary sleep state: wakefulness, slow-wave sleep (SWS), and rapid eye movement (REM) sleep. Each sample, chosen through meticulous visual inspection by trained observers, spanned a minimum duration of 30 seconds, ensuring a robust representation of the characteristic electrophysiological patterns associated with each state.

*Development and application of the KNN classifier*

Leveraging the capabilities of MATLAB (MathWorks Inc., Natick, MA), a K-Nearest Neighbors (KNN) classifier was developed to automate the categorization of subsequent 5-second data segments into one of the three predefined sleep states. The KNN algorithm, renowned for its simplicity and effectiveness in pattern recognition tasks, operates by comparing the feature vectors of new data segments against those of the visually selected reference samples. This comparison is based on the similarity of EEG and EMG patterns, with the algorithm assigning each segment to the sleep state of the closest matching reference sample.

### Statistical analysis

The impact of *G. biloba *extract on sleep parameters was statistically evaluated using one-way Analysis of Variance (ANOVA) to compare the total durations of REM, NREM sleep, wakefulness, and sleep spindle density across treated, sham-operated, and vehicle groups. Data compiled over a comprehensive forty-day period were averaged for each variable to ensure robust analysis. A *P*-value threshold of <0.05 was set to determine statistical significance, adhering to conventional criteria for biomedical research. This approach facilitated a rigorous assessment of the treatment effects, with all analyses conducted in compliance with best practices to ensure the validity and reliability of the findings.

## Results

### Drug-like properties of phytochemicals

Utilizing the SWISSADME computational platform (http://www.swissadme.ch), our analysis revealed that a substantial proportion of the screened phytochemical library exhibited promising drug-like characteristics. Specifically, 2299 out of the 2845 phytochemicals assessed were found to comply with Lipinski’s Rule of Five, underscoring their potential as viable candidates for further drug development endeavors. 

### Molecular docking

**Figure 2:**
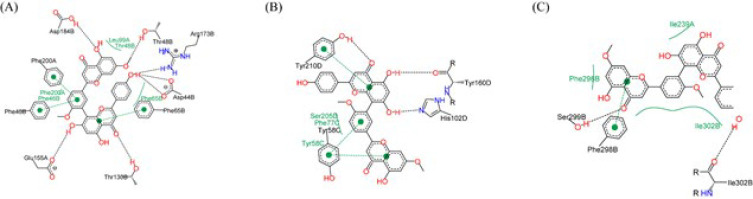
Molecular interaction profile of ginkgetin with GABAA receptor binding sites

**Figure 3:**
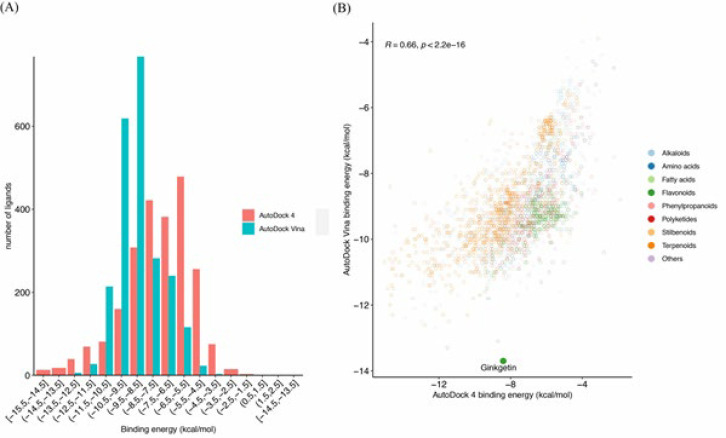
Correlation of predicted binding energies by AutoDock4 and Vina

Our molecular docking analysis, underpinned by rigorous self-docking validation, revealed that 8 out of 17 GABAA receptor structures achieved RMSD values below the 2 Å threshold, indicating high accuracy in our docking simulations (detailed in Table 1). Subsequent docking of the phytochemical library against these eight validated receptor models identified three structures, 6X3S, 6HUO, and 6CDU, as particularly conducive to high-affinity interactions. Table 2 presents the top 10 phytochemicals exhibiting the most favorable binding energies with these receptor models, all demonstrating binding affinities below -9 kcal/mol, indicative of strong potential interactions. Among the screened compounds, ginkgetin (PubChem ID: 5271805) emerged as a standout, exhibiting the lowest binding energy in the GABA binding site and ranking within the top 10 for both the benzodiazepine (BZ) and steroid sites. Notably, ginkgetin’s binding energy in the steroid site (-8.2 kcal/mol) closely approached the top values observed, underscoring its broad-spectrum affinity across multiple receptor sites. This compound’s superior biological activity, as documented in the PubChem and ChEMBL databases, warrants further exploration of its therapeutic potential. Visual representations of ginkgetin’s interactions within the GABA, BZ, and steroid-binding sites are depicted in Figure 2, providing insight into the molecular underpinnings of its binding efficacy. Additionally, Figure 3A illustrates the distribution of binding energies obtained from AutoDock4 (AD4) and Vina, with Figure 3B highlighting the significant correlation between the predicted binding energies from both docking programs (Pearson correlation: 0.66, *P*-value < 0.0001), reinforcing the reliability of our computational findings.

### Assessment of sleep parameters

**Figure 4:**
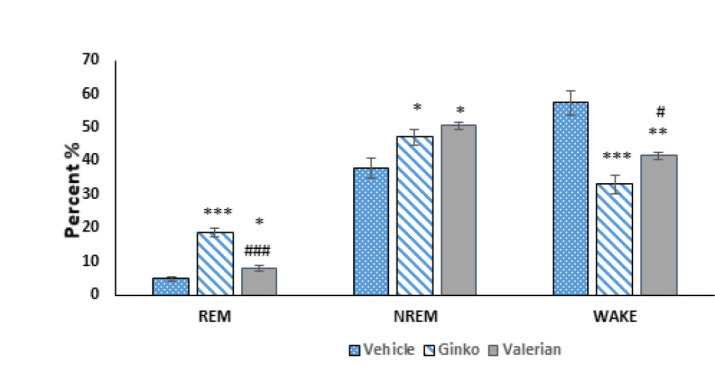
Comparative effects of* Ginkgo biloba *and valerian extracts on sleep architecture

**Figure 5:**
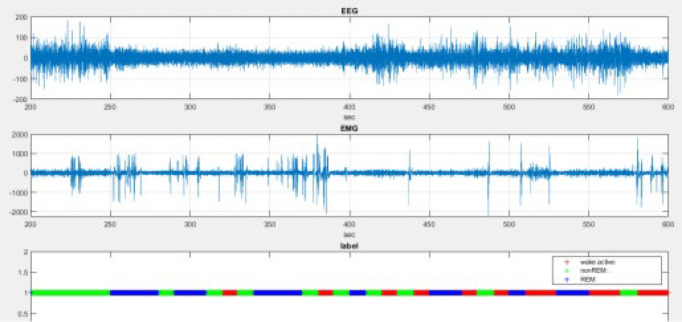
Hypnogram of *Ginkgo biloba* extract administration

 In our investigation into the effects of *G. biloba *extract on sleep architecture, a marked modulation of sleep states was observed. The cohort treated with *G. biloba* extract exhibited a pronounced reduction in wakefulness alongside a substantial increase in REM sleep, with the percentage of REM sleep escalating from 4.8% to 18.7% (*P*<0.0001), as illustrated in Figure 4. This contrasted with the group receiving Valerian extract, demonstrating a more modest increase in REM sleep of 8% (*P*<0.05). Notably, the decrease in wakefulness attributed to *G. biloba *extract was significantly greater than that observed in the Valerian extract group (*P*<0.05), further detailed in Figure 4. Moreover, both *G. biloba* and Valerian extracts were found to significantly enhance NREM sleep duration compared to the control group treated with solvent alone (*P*<0.05), with no significant difference detected between the effects of the two extracts on NREM sleep. The dynamic shifts in sleep stages, particularly the transitions into and out of REM and NREM sleep phases under the influence of *G. biloba* extract, are captured in the hypnogram presented in Figure 5. This visual representation underscores the profound impact of *G. biloba* extract on the sleep architecture of the treated animals, highlighting its potential therapeutic implications.

## Discussion

The exploration of phytochemicals for therapeutic applications has gained momentum. Our study focused on ginkgetin, a notable compound from *G. biloba*, for its potential in sleep modulation via the GABAA receptor. This research employed a multidisciplinary approach, integrating molecular docking with *in vivo *electrophysiological assessments, to elucidate ginkgetin’s effects on REM and NREM sleep. The findings demonstrate significant enhancements in sleep quality following *G. biloba* extract administration, positioning ginkgetin as a promising candidate for sleep disorder interventions.

The shift towards phytochemicals in sleep disorder treatment is driven by the limitations of conventional pharmacotherapy, including potential side effects and dependency risks. Phytochemicals such as melatonin and valerenic acid have established roles in sleep regulation, offering safer alternatives to traditional medications (20-23). Lavender essential oil, rich in linalool and linalyl acetate, has also been found to have sedative and anxiolytic effects (24). A study by Lin *et al*. (2015) showed that aromatherapy with lavender essential oil improved sleep quality in elderly participants with sleep disorders (25). Our findings contribute to this narrative, suggesting ginkgetin’s potential alongside these established sleep aids. Virtual screening (VS) in our methodology underscores the transformative impact of computational drug discovery, efficiently identifying therapeutic candidates like ginkgetin with high affinity for the GABAA receptor (26). Our* in vivo *findings supported this computational prediction, where *G. biloba* extract, enriched with ginkgetin, significantly altered sleep dynamics, notably increasing REM sleep duration. Ginkgetin’s influence on sleep likely stems from its multifaceted biological activities, including anti-oxidant, anti-inflammatory, and neuroprotective effects (27-32). These properties underpin its potential as a sleep modulator and suggest broader therapeutic applications. For instance, the neuroprotective attributes of ginkgetin may offer novel approaches to neurodegenerative disorders where sleep disturbances are prevalent (33). The enhancement of REM sleep, in particular, could have implications for cognitive health, given the role of REM sleep in memory consolidation and brain plasticity. However, the mechanisms underlying ginkgetin’s effects on the GABAA receptor and its broader biological impacts warrant further exploration. Future research should dissect these mechanisms at the molecular level, examining the interactions between ginkgetin and specific receptor subunits or signaling pathways. Additionally, the pharmacokinetics and pharmacodynamics of ginkgetin require thorough investigation to optimize its therapeutic application and ensure safety. The potential of ginkgetin to alleviate symptoms of neurodegenerative diseases opens up new research frontiers. The correlation between improved sleep quality and cognitive function suggests that ginkgetin could play a role in holistic treatment strategies that address both sleep and neurodegenerative symptoms. Moreover, the global burden of sleep disorders underscores the urgent need for innovative treatments. Sleep disorders, affecting millions worldwide, have been linked to a range of health issues, including cardiovascular diseases, obesity, diabetes, and mental health disorders (34-36). The economic impact of sleep disorders, through healthcare costs and lost productivity, further highlights the importance of effective interventions (37). In this context, phytochemicals like ginkgetin, with their potential for high efficacy and low side-effect profiles, represent a promising avenue for research and development. In addition to ginkgetin, other phytochemicals have shown potential in sleep modulation. Compounds such as apigenin, found in chamomile, have demonstrated anxiolytic and sedative effects, potentially contributing to sleep induction (38). Similarly, magnolol and honokiol, constituents of Magnolia bark, have been reported to possess anxiolytic properties, offering another avenue for sleep disorder treatment (39, 40). These findings underscore the vast potential of phytochemicals in sleep medicine, warranting extensive exploration. Exploring phytochemicals extends beyond sleep disorders, touching on various aspects of human health. For instance, the anti-inflammatory properties of curcumin, derived from turmeric, have been studied for their potential in treating chronic inflammatory conditions (41). Similarly, resveratrol, found in grapes and berries, has been researched for its cardiovascular benefits and potential to extend lifespan (42). These examples highlight the diverse therapeutic potential of phytochemicals, underscoring the importance of continued research in this field.

A) Stationary Phase: Silica geL 60 F 254 HPTLC Plate 5*10, Mobile Phase: Toluene-Ethyl acetate-Acetone-methanol (5-2.5-2.5-0.3), Derivatization reagent: Anisadehyde-H_2_SO_4_ (terpene-steroid). B) Stationary Phase: Silica geL 60 F 254 HPTLC Plate 5*10, Mobile Phase: Ethyl acetate-water-Formic acid-Acetic acid (10: 2.4 :1.1:1.1), Derivatization reagent: Natural Product (flavonoids, phenolic acid). C) Stationary Phase: Silica geL 60 F 254 HPTLC Plate 5*10, Mobile Phase: Ethyl acetate-water-Formic acid-Acetic acid (10: 2.4 :1.1:1.1), Derivatization reagent: DPPH. CH refers to the Chinese Ginkgo biloba sample, and IR refers to the Iranian *Ginkgo biloba* sample.

**Table 1:** Results of self-docking validation for GABAA receptor structures

PDB ID	Binding site	Self-dock RMSD	ΔG
6X3S	GABA	0.6	-11.6
6HUK	GABA	0.9	-11
6HUO	BZ	0.86	-11.5
6D6T	BZ	1.13	-9.4
6D6U	BZ	1.48	-9.6
6X3X	BZ	1.31	-9.5
6HUP	BZ	0.39	-10
6CDU	Steroid	1.38	-9.2

This table presents the receptor models achieving root-mean-square deviation (RMSD) values below 2 Å, indicative of accurate docking simulations. Binding energies (ΔG) are reported in kcal/mol, reflecting the affinity between each receptor model and its co-crystallized ligand.

**Table 2:** Summary of optimal docking interactions for phytochemicals at GABA, BZ, and steroid-binding sites of the GABAA receptor

#	PDB ID	Compound ID	Compound name	N-Cation-π	N-HB	N-HP	N-SB	N-TS	N-π-π	ΔG
1	6X3S	5271805	Ginkgetin	2	1	8	0	0	4	-13.7
2		160270	Dracorubin	1	1	7	0	0	4	-13.3
3		5281696	Sciadopitysin	2	2	9	0	0	4	-13.1
4		5462453	Glycobismine A	2	0	9	0	0	1	-12.7
5		442098	Tinyatoxin	0	1	12	0	1	0	-12.6
6		442082	C09179	0	1	12	0	1	0	-12.5
7		442369	Tiliacorine	0	1	8	0	0	3	-12.5
8		5281411	Toxiferine	1	3	7	0	0	2	-12.4
9		441805	Rutaevin	0	2	7	0	0	0	-12.3
10		443472	Neoabietadiene	0	0	7	0	0	0	-12.3
11	6HUO	11954224	C07544	0	1	9	0	0	2	-11.7
12		161671	Withanolide D	0	2	6	0	0	0	-11.6
13		5281809	Sojagol	0	0	8	0	0	2	-11.6
14		124069	Dihydrosanguinarine	0	0	7	0	0	1	-11.5
15		442328	Mecambroline	0	0	9	0	1	2	-11.5
16		442695	Blestriarene B	0	0	10	0	0	1	-11.5
17		5271805	Ginkgetin	0	1	7	0	0	3	-11.5
18		5281627	Hinokiflavone	0	1	11	0	0	2	-11.5
19		160503	Xylopine	2	0	8	0	0	2	-11.4
20		443774	Dioncophylline C	0	0	10	0	2	1	-11.4
21	6CDU	11953914	Spiredine	0	0	5	0	0	0	-9.4
22		442369	Tiliacorine	0	1	8	0	0	1	-9.9
23		161671	Withanolide D	0	0	5	0	0	0	-9.8
24		168919	Sempervirene	0	0	6	0	0	1	-9.8
25		5154	Sanguinarine	0	0	6	0	0	1	-9.8
26		5281386	C-Curarine I	0	0	10	0	0	0	-9.8
27		73062	Kaurenoic acid	0	0	8	0	0	0	-9.6
28		442980	Paravallarine	0	0	9	0	0	0	-9.5
29		265237	Withaferin A	0	1	5	0	0	0	-9.4
30		442728	Cassiamin C	0	0	5	0	0	1	-9.4

This table enumerates the top 10 phytochemicals exhibiting the highest binding affinities across the GABA, BZ, and steroid-binding sites, detailing the nature and number of molecular interactions involved, including cation-π interactions (N-Cation-π), hydrogen bonds (N-HB), hydrophobic contacts (C-C, N-HP), salt bridges (N-SB), T-stacking (face-to-face) interactions (N-TS), and π-π stacking (N-π-π). Binding energies (ΔG) are expressed in kcal/mol, underscoring the strength of each interaction.

This figure illustrates the specific interactions between Ginkgetin and amino acid residues within the (A) GABA, (B) BZ, and (C) steroid binding sites of the GABAA receptor, highlighting the intricate network of bonds and contacts that contribute to its binding affinity across these pharmacologically relevant sites.

GABA: γ-aminobutyric acid; BZ: Benzodiazepines

(A) displays the distribution of binding energies as predicted by AutoDock4 (AD4, red bars) and Vina (green bars), showcasing the comparative analysis across the docking platforms. (B) illustrates the relationship between the binding energies predicted by AD4 and Vina for phytochemicals interacting with the GABA binding site of the 6X3S receptor structure, highlighting a significant correlation (Pearson’s r = 0.66, *P*-value < 0.0001). The binding energy of Ginkgetin is specifically denoted with a green circle, emphasizing its notable affinity within the dataset.

This figure illustrates the differential impact of *G. biloba* and valerian extracts on REM sleep, NREM sleep, and wakefulness across treated and vehicle control groups. Significance markers indicate differences: ‘*’ denotes a significant deviation from the vehicle group, while ‘#’ highlights distinctions between the *G. biloba* and Valerian extract treatments. Significance levels are denoted as follows: * = *P*<0.05, ** = *P*< 0.01, *** = *P*<0.001.

This figure presents a hypnogram from an animal treated with a 200 mg/kg dose of *Ginkgo biloba* extract, showcasing the dynamic changes in sleep stages within the treated group over time.

## Conclusion

In summary, our study not only underscores the potential of ginkgetin as a novel agent for sleep modulation but also exemplifies the synergy between computational and empirical research in uncovering new therapeutic potentials. The significant impact of *G. biloba* extract on sleep induction adds to the growing evidence of phytochemicals as viable alternatives for sleep disorder treatments. The exploration of ginkgetin’s mechanisms of action and integration into treatment paradigms for sleep disorders and beyond necessitates comprehensive research.

## Declaration

We have not used any AI tools or technologies to prepare this manuscript.
